# Coupled Multiphysics Modelling of Sensors for Chemical, Biomedical, and Environmental Applications with Focus on Smart Materials and Low-Dimensional Nanostructures

**DOI:** 10.3390/chemosensors10050157

**Published:** 2022-04-25

**Authors:** Sundeep Singh, Roderick Melnik

**Affiliations:** 1 MS2Discovery Interdisciplinary Research Institute, Wilfrid Laurier University, Waterloo, ON N2L 3C5, Canada; ssingh@wlu.ca; 2 Schulich School of Engineering, University of Calgary, Calgary, AB T2N 1N4, Canada; 3 BCAM-Basque Centre for Applied Mathematics, E-48009 Bilbao, Spain

**Keywords:** coupled multiphysics models, low-dimensional nanostructures, sensors, heterostructures, carbon nanotubes, bandstructure calculations, smart materials, nanoplasmonics, graphene and carbon allotropes, metamaterials, first principles, atomistic-to-continuum methods, data-driven dynamic environments, biomarkers, rapid testing during pandemics, multiband Hamiltonians, wearable sensors, AI systems technologies

## Abstract

Low-dimensional nanostructures have many advantages when used in sensors compared to the traditional bulk materials, in particular in their sensitivity and specificity. In such nanostructures, the motion of carriers can be confined from one, two, or all three spatial dimensions, leading to their unique properties. New advancements in nanosensors, based on low-dimensional nanostructures, permit their functioning at scales comparable with biological processes and natural systems, allowing their efficient functionalization with chemical and biological molecules. In this article, we provide details of such sensors, focusing on their several important classes, as well as the issues of their designs based on mathematical and computational models covering a range of scales. Such multiscale models require state-of-the-art techniques for their solutions, and we provide an overview of the associated numerical methodologies and approaches in this context. We emphasize the importance of accounting for coupling between different physical fields such as thermal, electromechanical, and magnetic, as well as of additional nonlinear and nonlocal effects which can be salient features of new applications and sensor designs. Our special attention is given to nanowires and nanotubes which are well suited for nanosensor designs and applications, being able to carry a double functionality, as transducers and the media to transmit the signal. One of the key properties of these nanostructures is an enhancement in sensitivity resulting from their high surface-to-volume ratio, which leads to their geometry-dependant properties. This dependency requires careful consideration at the modelling stage, and we provide further details on this issue. Another important class of sensors analyzed here is pertinent to sensor and actuator technologies based on smart materials. The modelling of such materials in their dynamics-enabled applications represents a significant challenge as we have to deal with strongly nonlinear coupled problems, accounting for dynamic interactions between different physical fields and microstructure evolution. Among other classes, important in novel sensor applications, we have given our special attention to heterostructures and nucleic acid based nanostructures. In terms of the application areas, we have focused on chemical and biomedical fields, as well as on green energy and environmentally-friendly technologies where the efficient designs and opportune deployments of sensors are both urgent and compelling.

## 1. Introduction

Global challenges have accelerated the development of sensors in general and nanosensors in particular. The latter class of sensors is especially promising due to their small size, and hence easy integration into small units, as well as due to their high surface area resulting in significant signal changes, e.g., upon binding of an analyte or another object/substance. In this article our main focus is given to nanosensors and their applications, given the possibilities of their integration with nanoelectronics, enhancing further their processing capabilities. Specifically, we provide an overview of the most promising design platforms and modelling frameworks for such nanoscale devices that can measure physical quantities of interest, converting the result of measurements to signals which then can be detected and analyzed. Nanosensors are typically operating in a “label-free” regime, as long as labels (e.g., radioactive or fluorescent) are not used on the analytes/objects/substances being analyzed.

One of the most important classes of nanosensors is based on low-dimensional nanostructures which include, but are not limited to, nanowires, nanotubes, quantum dots, and nanoribbons. The development of such nanosensors, their geometry, and the materials to be used are dependent on their areas of application. For example, for nano-force and nano-mass measurements, CNT-based nanosensors could be advantageous compared to other material usages (e.g., silicon or ZnO), while ZnO nanowires could be extremely useful for gas sensing applications, due to their easy and low-cost fabrication, together with high sensitivity toward low concentrations of gases under ambient conditions.

We focus here on two classes of materials due to their great potential in sensor technologies, low-dimensional nanostructures, and smart materials. The first class covers heterostructures that already have taken their firm place in sensor applications, as well as emerging fields such as nucleic-acid-based nanostructures due to rapid advances of the architectonics of programmable molecules such as RNAs and DNAs. The second class covers more traditional materials such as shape memory alloys as well as new developments in smart materials and structures technologies. We focus on the modelling issues of the devices, applicable in sensing, from the choice of materials, properties, and designs to their utilizations and operation in larger units. As a result, the major emphasis is given to multiscale models capable of integrating coupled physical effects, essential in such cases, e.g., thermomechanical, electromechanical, etc. In its turn, it requires the development of efficient envelope-function, coarse-grained, and other approximate models which are discussed here, along with reduced-order models and efficient numerical procedures for their implementations. Most applications discussed here are pertinent to global challenges faced by our world today in biomedical, chemical, and environmental fields and to those that will remain relevant for the next decades.

The rest of the paper is organized as follows. In Section 2, we emphasize the importance of the development of sensors based on low-dimensional nanostructures, while Section 3 focuses on smart materials for sensing applications. Section 4 is devoted to mathematical and computational models for the main two classes of materials discussed in the previous two sections. The cornerstone of this section is in coupled multiscale models, limitations of first-principles approaches, and the importance of reduced-order and approximate models capable to capture the main aspects of the dynamics and physics of the underlying processes. Properties of nanostructure-based sensors are also discussed in this section on the examples of strain and pressure sensing applications. Section 5 is dedicated to several important classes of novel sensing applications, including those based on carbon allotropes and nucleic acids, that came to prominence due to the emerging field of architectonics of programmable molecules. An important part of this section includes also sensing applications for environmentally-friendly technologies, including green energy and lead-free economic trends. The section is closed with several examples of wearable sensors important for future AI systems technologies, among many other current and potential applications. Conclusions are given in Section 6.

## 2. Low-Dimensional Nanostructures and Sensors

**Figure 1:**
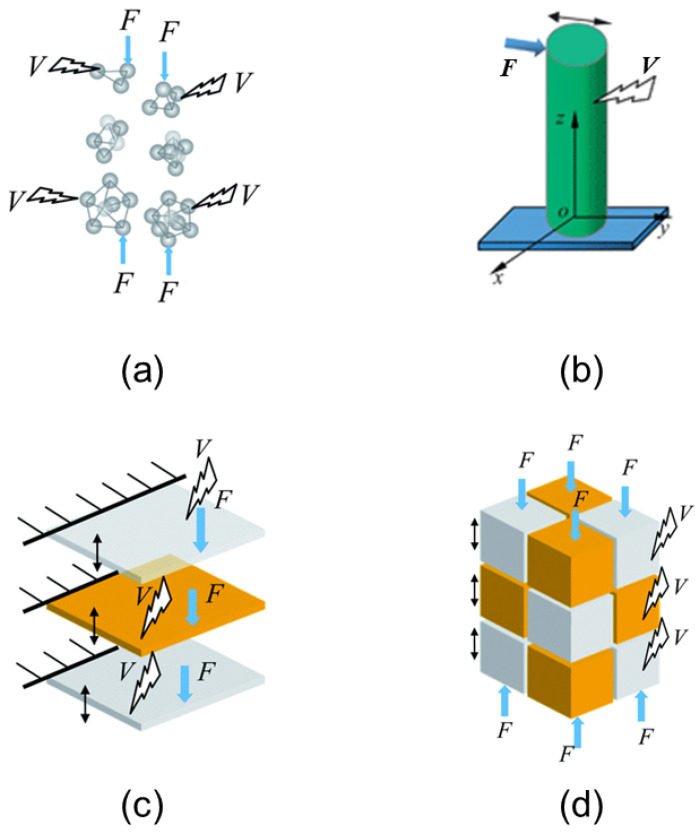
Schematic view of different dimensions of piezoelectric nanostructures: (**a**) zero-dimensional (e.g., nanocluster materials and nanodispersions), (**b**) one-dimensional (e.g., nanowires, nanotubes and nanorods), (**c**) two-dimensional (e.g., nanoribbons and nanofilms), and (**d**) three-dimensional (e.g., piezoelectric powders, fibrous, multilayer and polycrystalline materials where the 0D, 1D and 2D structural elements are in close contact with each other). (This figure is reproduced with permission from [1]).

Low-dimensional nanostructures (LDNs) play a prominent role in developing innovative sensors, as well as in endowing the sensors with new enhanced functional capabilities. This can be said for all classes of such nanostructures, which are classified depending on the number of directions where the motion of carriers is confined, resulting in the discretization of energy levels. The latter fact leads to a situation where low-dimensional nanostructures have a sharper density of states compared to structures of more conventional sizes. Typical examples include two-dimensional nanostructures (2D) such as nanosheets and nanowalls, thin quantum films and wells, one-dimensional (1D) such as nanowires and nanorods, nanotubes and nanoribbons, as well as zero-dimensional (0D) such as quantum dots, nanoparticles, and nanoclusters. Figure 1 presents the schematic views of different dimensions of the piezoelectric nanostructures.

Low-dimensional nanosystems, such as quantum dots, have been playing an important role in peptide science, including sensing applications. In particular, peptides can serve for tagging protein ligands and biosensors to QD surfaces (e.g., [2]). Among LDNs, nanowires play a very important role in developing a wide range of sensors. There are also expectations that nanowires will lead to significant progress in biomedical sensors, where their applications range from the detection of biomarkers, viruses, and DNAs to drug discovery. This should not come as a surprise given that the size of many biomolecules is on the order of 10–100 nm, putting them in the same range as synthetic nanostructures. There are also arguments that, unlike CNT-based biomedical sensors which require further purification after their synthesis from mixing semiconductors and metals, nanowire’s surface can be modified straightforwardly to become sensitive to chemical and biological species [3]. As an example, among other LDN-based sensors, we mention luminescent chemosensors that have been developed for various purposes in biomedical research such as monitoring DNA hybridization, detecting small molecules, and assessing proteins (e.g., [4]).

Starting from the quantum Liouville equation [5], a hierarchy of approximations for the analysis of low-dimensional nanostructures can be constructed based on mathematical models derived from bottom-up and top-down approaches in a way similar to the classification of self-consistent models in quantum mechanics describing open quantum mechanical systems with relaxation mechanisms [6]. In the latter case, one starts from von Neumann–Poisson (or quantum Liouville–Poisson) model and carries on with relaxation-time approximations. The resulting classification has played an important role in the modelling of quantum systems, in particular semiconducting, by using the Wigner-Poisson mathematical framework. The far-reaching influence of this approach is apparent today in developing efficient numerical approaches such that the von-Neumann model in center-of-mass coordinates would lead to the quantum Liouville-type model, once a suitable basis is chosen and the Wigner formalism is used, providing a rich foundation for phase-space formulations [7]. The state of a nanostructure, having *n* degrees of freedom, can be described by utilizing the Wigner distribution function ρ (the density operator) in the Wigner–Weyl phase space. Let *q* and *p* be the vectors representing the *n* coordinate and *n* momentum operators, respectively. Then, assuming the system motion owing to a Hamiltonian function H(q,p,t), it is well known (e.g., [8]) that the evolution of the state of the system can be described by the quantum Liouville equation:

(1) $$i\hbar \frac{\partial \rho }{\partial t}=\left[H,\rho \right]=H\rho -\rho H.$$

At the same time, it is often more convenient to use a generalized form of the quantum Liouville equation where the Hamiltonian operator:

(2) $$H=\sum_{i=1}^{n}\frac{p_{i}^{2}}{2m_{i}}+V\left(q,t\right),$$

is split into two Hamiltonians describing the nuclear motion for the lower and upper electronic states, as:

(3) $$H_{l}=T+V_{l};\;H_{u}=T+V_{u},$$

where $V_{l}$ and $V_{u}$ are the effective potentials of the nuclei in the lower and upper electronic states, respectively, and *T* is the kinetic energy. Among other applications, this generalized approach is important for calculating molecular (vibrational and electronic) spectra (e.g., [9]), and more generally, for a diverse range of problems involving systems at finite temperatures, open quantum systems, and dissipative environments [10,11]. Taking into account (2)–(4), the generalized quantum Liouville equation then becomes:

(4) $$i\bar{h}\frac{\partial \rho }{\partial t}=H_{u}\rho -\rho H_{l}.$$

This equation has the form of a time-dependent Schrödinger equation for a quantum system with N=2n degrees of freedom. A nonrelativistic time-independent version of this equation is presented as:

(5) $$\hat{H}\Psi =E\Psi ,$$

where *E* represents the total energy of Hamiltonian operator $\hat{H}$ and Ψ is the wavefunction.

The approach based on the quantum Liouville model, as presented above, has its roots in the Wigner–Weyl isomorphism between operators and symbols, providing a comprehensive phase space representation of dynamics [12,13]. Note also that within the Wigner–Weyl phase space formulation of quantum mechanics, a version of the uncertainty principle can be derived revealing deep interconnections between geometry and uncertainty [14], while the approach itself provides a viable link between probabilistic mechanics and quantum theory [15]. When dealing with applications of open quantum systems where we have to account for the influence of the external environment, the density matrix tools have been popular for a long time (see, e.g., [16,17,18,19,20,21,22,23] and references therein), including sensor applications [24]. As mentioned, the primary step in the approach advocated here is the density operator, where the density matrix is its possible representation that allows to describe the state of a quantum system and to calculate the probabilities of measurement outcomes carried out upon the system, once the Born rules are imposed. The approach to the analysis of open systems based on the quantum Liouville model continues to be under active developments [25,26] with advanced applications stimulating further progress in phase space formulations [27], including the Wigner–Weyl formulation as highlighted above.

Low-dimensional nanostructures are often made from alternating layers of different semiconductors, producing heterostructures [28,29,30]. Many interesting proposals, explored in the past, have been related to the modelling of quantum heterostructures for sensing applications [31], with the list has been continued to grow, including the research on quantum sensors in single layer 2D materials with high sensitivity [32]. Exploration of new material solutions towards atomic-scale sensing in low dimensions is an active area of research today. One of the most important representatives of 2D materials is graphene and its composites. Hence, it should not come as a surprise that various types of sensors are being investigated based on graphene heterostructures. An interesting example of linear piezoresistive strain sensors has been recently considered in [33]. With a combination of 2D graphitic carbon nitride and graphene heterostructure, the authors have been using a novel hybrid material that exhibits piezoresistivity superior to graphene. The desired effect has been due to the 2D graphitic carbon nitride interface with periodically spaced triangular nanopores allowing the improved piezoresistivity of the sensor, achieved by transmitting the change in polarization once the strain is applied. Such interfaced materials are difficult to analyze experimentally, as well as computationally. Although density functional theory (DFT) studies are computational expensive and usually inaccessible at the device level, they could provide important initial insights into the underlying mechanisms such as the reported strain-dependent band gap opening which occurs in the graphitic layer of the composite materials under strain [33]. These new observations point out towards prospects of these novel materials in strain pressure sensor applications.

When dealing with low-dimensional nanostructures and systems, one of the key challenges lies in the fact that structural and electronic properties of these materials are interconnected and geometry-dependent [34,35,36,37,38]. As a results, an important insight into such properties used for designing the devices such as sensors are provided via bandstructure calculations. This can be done with several numerical methodologies discussed in more detail in Section 4.2, subject to our choice of the basis functions for the Schrodinger-type model. The design of low-dimensional-nanomaterial-based sensors is a multiscale problem, more complex than the multiscale modelling of nanostructures themselves [39], requiring the integration of coupled effects into the model (e.g., electromechanical interactions, etc.). Subsequently, among the most common semi-empirical numerical approaches, the k·p method has been demonstrated to be robust, reliable, and computationally efficient, in particular for low-dimensional semiconductor nanostructures [30,40,41]. The construction of multiband Hamiltonians for this method in solving coupled multiscale problems is closely interwoven with some of the fundamental questions of quantum mechanics and mathematical physics [11] which are becoming increasingly important for the development of data-driven models.

In the meantime, substantial progress has been made over the recent years in the application of ab initio studies in determining geometry-dependent properties of low-dimensional nanostructures important for sensors [42,43,44]. Some recent examples of this type include nanowire-based sensors [45], specifically gas/chemical sensors [46], photodetectors [47], and (field-effect transistor) FET-sensors [48]. Geometry-dependent properties of CdS nanowires with target applications in solar cells and gas/chemical sensors have been studied ab initio in [49]. The geometries, analyzed with DFT, included wurtzite hexagonal and triangular shape nanowires, which revealed increased stability with enlarging diameter. While this latter characteristic reduces the bandgap, it increases the electronic charge carrier mobility. Further, geometry-dependent properties have also been demonstrated with the DFT approach applied to the analysis of CdSe nanowires in their wurtzite phase [50]. From a computational point of view, the corresponding DFT model was used under the standard Perdew Burke and Ernzerhof parameterization, with localized double-zeta polarized orbital basis set, and the generalized gradient approximation. Note also that chemical and biological sensors developed based on CdSe nanostructures have a long history (e.g., [51]).

The development of sensors based on one-dimensional nanostructures such as carbon nanotubes (CNTs), various nanowires (e.g., silicon, palladium), as well as polymer and metal-oxide-based nanofibers, has been under the active radar of researchers since the beginning of this century. More recently, nanoribbons have been investigated in sensors, and exciting new developments include also nanoribbons based on 2D materials (e.g., MoS${}_{2}$ and MXenes) which have rapidly become a new building element for sensor designs. We should also mention that hybrid systems, metamaterials, and various hierarchical materials (e.g., with additions made to CNTs or nanowires to increase sensitivity) represent another important avenue of recent endeavors. Superconducting nanowire-based sensors have also been a subject of intensive investigations [52,53,54,55,56]. While new nanomaterials continue to be developed and analyzed, the issues of their synthesis and fabrication (along with the processes for preparing such nanomaterials for sensor applications) are outside the scope of this paper, and the interested reader may consult the relevant literature on these issues, such as an excellent recent review [57].

Next, we would note that zero-dimensional nanostructures have also been playing an important role in developing innovative sensing technologies. One of the examples in this direction includes longwave-infrared remote sensing and spectral imaging devices, in particular the development of quantum-dot infrared photodetectors [58,59]. As in the number of cases already considered by us, it should be emphasized that the knowledge of bandstructure here is critical too, because, e.g., the bias-dependent spectral response of these devices is brought about by the asymmetric bandstructure of the dot-in-a-well configuration. Clearly, for quantum dots, the calculation of bandstructure is a more involved process requiring careful approximations of multiband Hamiltonians and other considerations [60,61,62,63,64,65,66,67]. In the specific case of the applications we mentioned, the photocurrents, driven by different operational biases, are viewed as outputs of different bands. Other challenges with quantum-dot infrared photodetectors are their significant spectral overlap and detector noise, which both complicate feature selections. Therefore, the development of spectral sensors in this area requires stochastic mathematical models and Bayesian approaches. Recently stochastic methodologies have also been applied to the analysis of graphene nanoribbons with several machine-learning-based techniques [68,69]. Various carbone allotropes (graphite—3D, Graphene-2D, CNT-1D, Fullerene 0D, Diamond 3D, and graphyne [35,70,71,72,73,74]) can lead to instructive and potentially viable applications, with graphene-based sensors leading the way in many areas, including human health [75], which we discuss further in Section 5.1. Moreover, due to its prominence in sensing applications, we shall return in that section to another group of these allotropes, namely CNTs. There is also increasing attention to novel compounds such as nanodiamond-nanotube composite materials, largely because we know that diamond can assist in revealing the movement of cells around the body, delivering genes, drug targeting, molecular machines. Hence, the applications of nanodiamond in disease detections, human blood samples highlight its increasing role in bionanotechnology, including sensing. The knowledge on electronic structure and transport properties of low-dimensional nanostructures is also important for control, e.g., electric and acoustoelectric, as well as control via spin, with coupling phenomena, such as spin-orbit coupled effects, considered to be very important [76], especially given new horizons and potential of spintronics. Among other coupled effects we will also mention here those induced by nonlinear acoustoelectric transport where we have to model, for example, the interactions between surface acoustic waves (SAWs) and carriers in low-dimensional nanostructures. In such cases, we can utilize the fact that electrons can be carried by sound waves for future device implementations and designs while bearing in mind that the range of applications of SAW sensors is already very impressive [77].

**Figure 2:**
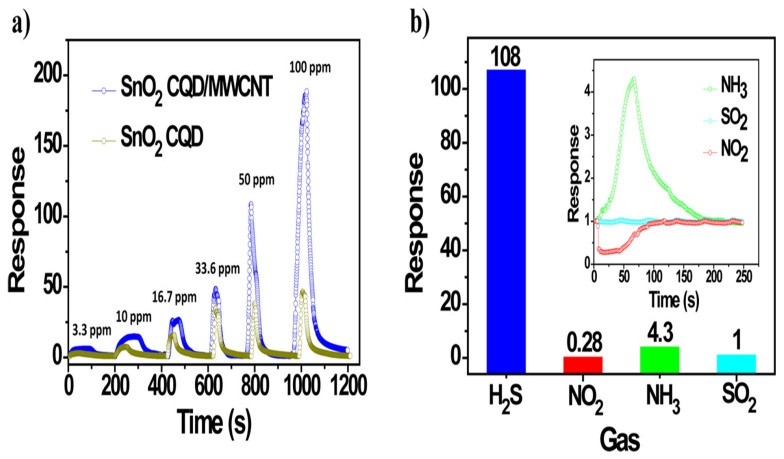
(**a**) Response curves of the sensors based on SnO${}_{2}$ colloidal quantum dots (CQD)/multiwalled carbon nanotubes (MWCNT) nanocomposites and pristine SnO${}_{2}$ CQDs upon H${}_{2}$S exposure/release cycles at 70 ${}^{\circ }$C. (**b**) Selectivity of the SnO${}_{2}$ CQD/MWCNT gas sensor at 70 ${}^{\circ }$C. (This figure is reproduced with permission from [81]).

Finally, in the remainder of this section, we will focus on metal oxide-based sensors which are important in environmental applications and gas sensing where ZnO, in particular, plays an important role. We note that many nano-heterostructures applied in sensors are metal oxide-based. Due to their low cost, high sensitivity, and environmentally friendly fabrication, one of the main emphases in the literature has been given to resistive-type gas sensors [78]. The key mechanism of their functioning, based on electrical resistance measurements to detect the presence of gas, has been known for a long time. However, the current improvements in sensitivity and selectivity have become possible with the advent of nano-heterostructures. Advantages also include the increased adsorption and the possibility of creating a charge carrier depletion layer that produces a larger modulation in resistance, as well as accelerated catalytic activity. From a modelling point of view, the analysis of such sensors is based on coupled models, accounting for the combined synergetic effects acting to amplify the reception and transduction of the sensor signal. The building block used for the design of such gas sensors is often a low-dimensional zinc oxide (ZnO) structure with coating, e.g., a zeolitic imidazolate framework (ZIF) coated ZnO nanorod [79]. In this case, the heterostructure has a ZnO core and a ZIF shell. Modelling of such core-shell heterostructures is often done by using finite element analysis, aiming at designing the structures with enhanced selectivity of gas sensing materials. Another class of ZnO-based heterostructures for gas sensing includes branched heterostructural composites such as those that use ZnO nanorod backbones and SnO${}_{2}$ branches [80] which were tested on ethanol response, demonstrating superior characteristics compared to pure ZnO gas sensors. Figure 2a present the sensitive and fast recovery response of the gas sensors based on the SnO${}_{2}$ colloidal quantum dots (CQD)/ multiwalled carbon nanotubes (MWCNT) nanocomposites and pristine SnO${}_{2}$ CQDs upon H${}_{2}$S exposure/release cycles at 70 ${}^{\circ }$C. Figure 2b depicts the comparison of the SnO${}_{2}$ CQD/MWCNT gas sensors toward several hazardous air pollutants.

## 3. Smart Materials for Sensing Applications and Coupled Effects

First of all, it should be noted that various heterostructures discussed in the previous section are based on smart materials and that the development of low-dimensional nanostructures and smart materials often go hand in hand in applications of sensor technologies. One of such classes includes multiferroic heterostructures that can be synthesized by integrating monolithic ferroelectric and magnetic materials [82]. Such integration includes interfacial coupling between electric polarization and magnetization which is manifested via elastic, electric, and magnetic energy exchange. It is well known that this cross-coupling can be controlled via the direct or converse magnetoelectric effects, varying the magnetization (or polarization) with an electric (or magnetic) field. As a result, there is a potential in improving and/or adding new functionalities to some of the existing and emerging sensors. At the same time, much about the nature of the interfaces and interfacial coupling remains to be explained and the modeling plays an important role in this.

Among other groups of materials, we will mention shape memory alloy (SMA) nanostructures which allow building novel functionalities based on thermo-mechanical nonlinear effects [83]. While dealing with this type of materials, some of the challenges we face include size effects (e.g., critical dimensions), boundary conditions, as well as complex multidimensional dynamics of the underlying phenomena. Many of these challenges have been effectively addressed with novel 3D models based on coupled dynamic phase-field theories [84]. Another group of materials important for sensor applications includes ferroelectrics. Already for several decades bulk ferroelectric materials have been enthusiastically used in sensor and actuator technologies. A spontaneous polarization, observed below the Curie temperature, is typically responsible for that as it is quite sensitive to external stimuli such as mechanical, electric, thermal fields, chemical, and biological factors. However, better sensing properties can frequently be obtained with ferroelectric nanostructures, and the range of the application of the latter class has been growing steadily, including nowadays gas sensors, photo- and radiation detectors, various thermomechanical sensors, unitizing piezoresistivity, piezoelectric, and pyroelectric coupled effects, as well as biosensors. The recent achievements and known challenges in this area are closely related to unraveling the phenomena intrinsic to the dynamics of ferroelectrics which have been studied extensively with the models based on the Landau theory [85].

The application of SMA-based systems and devices in sensor and actuator technologies is well documented, including the areas of biomedicine, robotics, the automotive, and aerospace industry. As an amalgamation of metals that can remember their shape, they can be made from copper-aluminum or nickel-titanium alloys, as well as from other elements such as zinc, gold, and iron. Each class has its advantages, e.g., copper-based SMAs are characterized by low cost, while nickel-titanium SMAs demonstrate superior thermo-mechanic properties. As a result, SMAs and similar nonlinear materials offer a plethora of options for the intelligent design of sensor-actuators, but their dynamics with solid-solid phase transformations and sophisticated microstructure evolutions represent a serious challenge in modelling, and innovative numerical methodologies are required [86,87,88,89,90,91,92,93,94,95,96,97,98,99,100,101,102,103,104,105,106,107,108,109,110]. As these materials change shape in response to temperature, high forces and significant motion can produce a substantial work output. This, together with their fast response and small size in modern designs, lead to their applications in thermal compensation and force sensing. In biomedicine, these materials are important in minimally invasive surgery and therapy not only as actuators, but also as strain sensors important for flexible surgical and intervention instruments, drug delivery systems, and osteotomes devices, etc. Their lightweight, biocompatibility, and low cost are clear indications that their importance will continue to grow. Among others, the area of minimally invasive surgery and therapy drives the device size reduction and the development of adequate sensing technologies where unique features of SMAs some of which we already mentioned (bio-compatibility, low cost, lightweight, large actuation forces, and electrical resistivity variations) poise these materials for success [111]. Other innovative areas for SMAs continue to be automotive and aerospace industries, with many examples such as environmental pressure sensors for combustion monitoring and control in cars.

**Figure 3:**
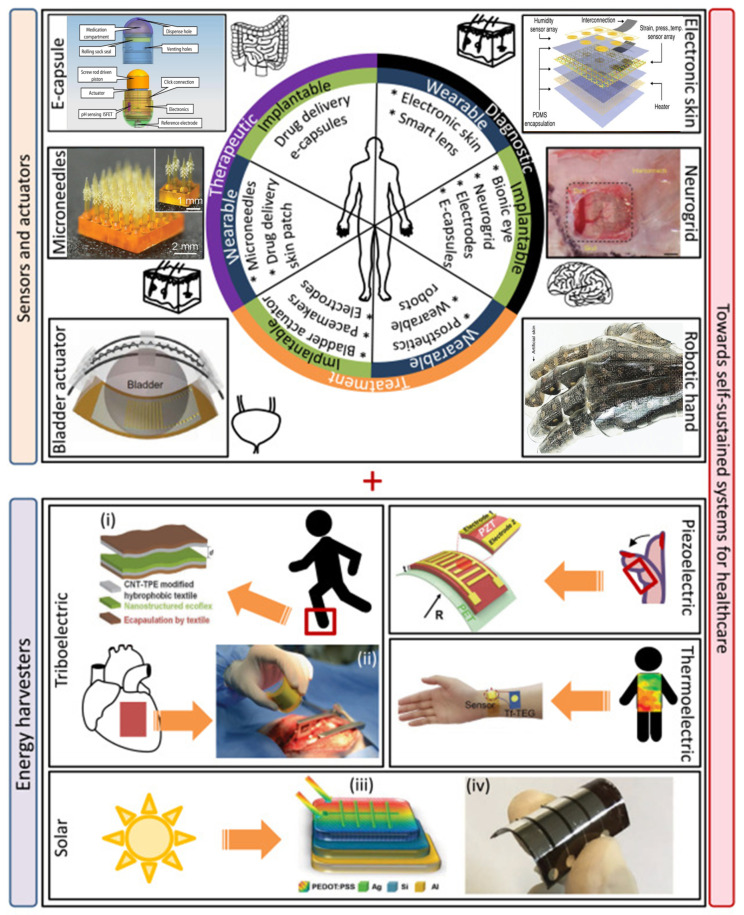
Wearable and implantable devices on/in human body, viz., E-capsule, electronic skin, microneedles, neurogrid and robotic hand. Self-sustained systems for healthcare, viz., wearable triboelectric energy harvester (**i**), implantable triboelectric energy harvesters (**ii**), piezoelectric energy harvester, thermoelectric energy harvester and solar energy harvester (**iii**,**iv**). (This figure is reproduced with permission from [118,119,120,121,122,123,124,125,126,127,128,129,130]).

Over the past several decades, SMAs have driven advances in many important areas, including biomedicine, microelectronics, and energy conversion. These advances, linked to the extraordinary progress of nanoscience and nanotechnology, demonstrate a clear tendency towards further miniaturization [112]. As a result, further developments of novel dynamic models describing coupled nanomechanics of various types of these materials [83,84,113,114], including NiTi-based and Cu-based shape memory alloys, become increasingly important. Furthermore, innovative designs of hierarchical nano-assemblies of functional nanoscale/meso/macroscale devices open up new perspectives for the creation of novel systems for numerous applications. Along this avenue, CNTs, InP, ZnO nanowires and nanoparticles, and SMAs materials can play a pioneering role in designing biomedical nanosensors and other applications [115]. A subset of applications of smart materials in healthcare is presented in Figure 3. This subset provides examples covering different areas of healthcare such as surgery, diagnostics, monitoring, rehabilitation, as well as drug delivery tools, and self-sustained energy systems, applied in wearables and implantable devices [116,117,118,119].

## 4. Mathematical and Computational Models for Smart Materials and Low-Dimensional Nanostructures

In this section, we provide a more detailed description of complementary tools to experiments that are becoming increasingly important in the modern design, analysis, and optimization of sensors and material components on which they are based. We start from the applications where sensing such physical characteristics as strain, pressure, mass, and force is critical. These applications provide an important motivation for the development of coupled multiscale models where the interactions between different physical fields are systematically accounted for. In the case of the development of nanosensors, this includes also quantum mechanical models where bandstructure calculations become paramount for predicting nanostructure properties. In discussing the limitations of first-principles approaches, we emphasize the importance of developing accurate approximate models, allowing seamless integration of coupled processes throughout the scales involved. In this context, the k·p approach and envelope-function approximations are discussed, along with the derivation of multiband Hamiltonians based on systems of coupled Schrödinger-type equations. The role of nonlinear effects, in particular those induced by the geometry of the problem, as well as of nonlocal phenomena, is emphasized. Furthermore, in some cases, a specific geometry of the problem can substantially reduce computational costs. However, this requires additional efforts at the stage of the construction of multiband Hamiltonians [131,132], e.g., when we deal with cylindrical nanowires, as well as in the case of barrier localization and critical radius limitations [133]. Spin-orbit interactions in three-dimensionally bounded nanostructures is another important problem to be addressed in such cases [134], whereas possibilities for plasmonic applications arising from low-dimensional nanostructure defects and other processes and phenomena [135,136,137] can represent substantial interest for future applications. In many applications, in particular, those based on smart materials such as shape memory alloys, we need to deal with complex nonlinear dynamic processes connected with first-order solid-solid phase transitions and microstructure evolutions. As a result, the concluding part of our discussion in this section deals with these problems, focusing on time-dependent models. Computationally complexity in such cases dictates the development of atomistic-to-continuum models, reduced-order methods, and coarse-graining, which rely on efficient numerical procedures which are discussed here.

### 4.1. Coupled Multiscale Models: Strain and Pressure Sensing Applications

**Figure 4:**
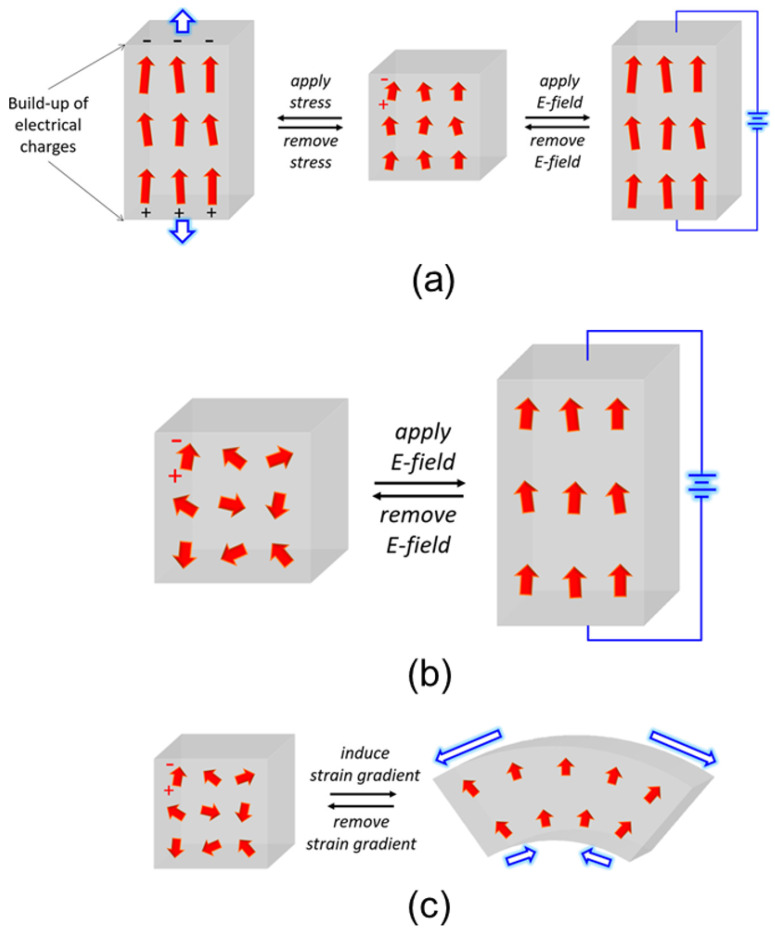
Schematic illustration of: (**a**) direct (**left**) and converse (**right**) piezoelectric effects, (**b**) electrostriction, and (**c**) flexoelectricity. The red arrows represent electric dipoles, and the blue arrows represent the applied mechanical stress. Piezoelectricity is the linear electromechanical coupling between strain and electric field, whereas electrostriction is the nonlinear (quadratic) coupling between strain and electric field. Flexoelectricity is a consequence of the linear coupling between the electric field and strain gradients. The direct piezoelectric effect refers to an electric polarization induced by an applied mechanical stress, while converse piezoelectric effect relates a physical strain induced under an applied electric field. Piezoelectricity requires lack of inversion symmetry or noncentrosymmetric arrangement of dipole moments. When the red arrows (that represent dipole directions of noncentrosymmetric domains) are preferentially aligned along one direction, the material can exhibit piezoelectricity. Unlike piezoelectricity, electrostriction does not require an alignment of permanent dipoles across all domains. In contrast to piezoelectricity that is produced by uniform strains, flexoelectricity is a size-dependent electromechanical coupling phenomenon that appears from inhomogeneous strains or a strain gradient. (This figure is reproduced with permission from [144]).

The quest for ultrasensitive, fast, portable, and robust pressure and strain sensors, capable of operating in various harsh environments, has been growing consistently over the past several decades. In this context, a major emphasis has been given to the materials that exhibit beneficial stabilities (e.g., thermal, mechanical, chemical), as well as radiation hardness [31]. Given that heterostructures develop sheet charges at the heterointerfaces due to spontaneous and piezoelectric polarizations, they have played an especially promising role here. Heterostructures can also be designed in such a way that they minimize known problems, including those coming from the undesirable charge carrier generation (optical and/or thermal) due to large bandgaps and atomic bondings. However, when such structures are to be used in pressure or stress sensing, a detailed analysis of the stress-induced modulation of the barrier height is required. Moreover, due to the lattice-mismatched, strain comes naturally as one of the crucial parameters to account for, while the temperature may also play a decisive role. As a result, at the modelling level, we are led to a coupled model which describes thermopiezoelectric effects in nano-sized heterostructures and quantum nanowires [138,139,140], which in some cases should be extended to account also for higher-order nonlinearities as well as nonlocal effects [141,142,143]. Figure 4 presents the schematic illustrations of the intrinsic material processes, i.e., electromechanical coupling responses that are related to the crystal symmetry and molecular structure of the material, viz., piezoelectricity, electrostriction, and flexoelectricity.

The electromechanical couplings between the strains, strain-gradients, and electric fields are introduced in a free-energy density function utilizing a thermodynamics-based constitutive framework. Let *T*, *S*, ϵ, σ, *P*, and *E* be the temperature, entropy, strain, stress, polarization, and electric field, respectively. Then, the Gibbs free energy function which includes all these couplings is given as follows:

(6) $$\begin{array}{c} dG=-S\cdot dT-\epsilon_{ij}\cdot d\sigma_{ij}-P_{k}\cdot dE_{k},\\ \sigma_{ij}={\left(\frac{\partial G}{\partial \epsilon_{ij}}\right)}_{P,S};\;E_{i}={\left(\frac{\partial G}{\partial P_{i}}\right)}_{\epsilon ,S};\;T={\left(\frac{\partial G}{\partial S}\right)}_{\epsilon ,P}. \end{array}$$

**Figure 5:**
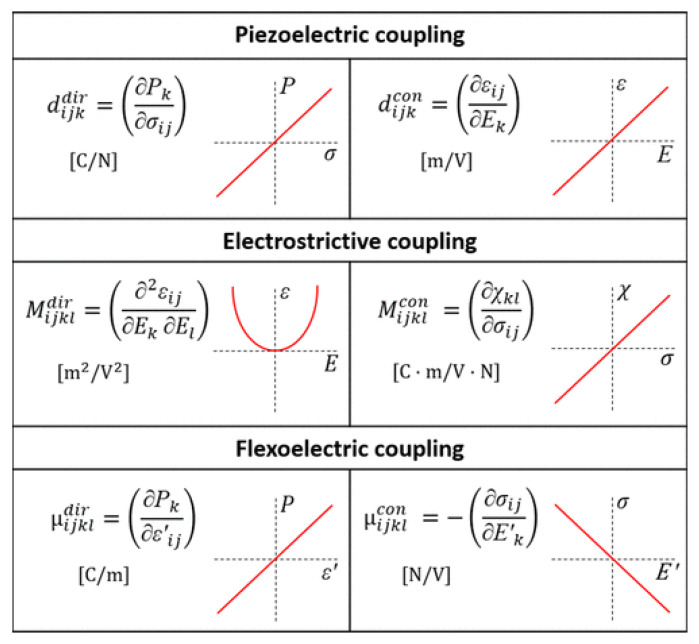
Relationships between mechanical variables and electrical variables in the piezoelectric, electrostrictive, and flexoelectric couplings. $\epsilon {}^{\prime }$ and $E{}^{\prime }$ in the flexoelectric coupling represent the strain gradient and electric field gradient, respectively. (This figure is reproduced with permission from [144]).

These electromechanical couplings are represented by piezoelectricity, electrostriction, and flexoelectricity that couple mechanical (strain and stress) with electrical properties (polarization, electric field, and dielectric displacement or flux) in different ways. Each coupling mechanism brings about a characteristic electromechanical response. By using conventional notations for mechanical variables (strain (ϵ) and stress (σ)) and electrical variables (polarization (*P*), electric field (*E*), and dielectric susceptibility (χ)), we can see such responses in Figure 5 for both the direct (dir) and converse (con) coefficients of each coupling.

The piezoelectric effect continues to be a key element in the development of the associated coupled models as it is widely employed in pressure sensors, for example, for the detection of dynamic signals. However, several known challenges exist for such piezoelectric-induced pressure sensors. For example, if the measurements are based on the transient flow of electrons in an external load (arising from the dynamic-stress-generated piezo-potential), then the measurements of static signals can be a challenge. Today, with piezoelectric nanowires/graphene heterostructures employed for static measurements, these challenges can be overcome by using combined mechanisms between (a) strain-induced polarization charges in piezoelectric nanowires and (b) the resulting change of carrier scattering in graphene heterostructures [145]. With these recent discoveries, such flexible piezoelectric-induced pressure sensors can be applied in electronic skin and wearable electronics, e.g., in wearable sweat sensors which pave the way for real-time analysis of body chemistry (see also Section 5.3).

Low-dimensional carbon nanomaterials, such as carbon nanotubes, graphene sheets, and carbyne, also play an important role in designing various types of nanomechanical sensors due to their excellent mechanical, thermal, and electrical performance. Among promising directions, we point out here the development of carbon nanomaterials-based nano-force and nano-mass sensors which have demonstrated better performance, in particular in their higher sensitivity, compared to the devices based on silicon and ZnO [146]. The latter work has included also mathematical models for frequency-based nanosensors and a possible extension of such models to carbon-based sensors, keeping the simplicity of the continuum-based approach for the mechanical analysis of the behavior of their resonators. In better understanding sensing mechanisms, mathematical and computational models are playing an increasingly important role, and this includes funtionalized nanowire sensors such as those based on functionalized ZnO nanowires. More generally, the development of micro and nanomechanical resonators can be viewed as part of the advancements made in micro-electromechanical systems/nano-electromechanical systems (MEMS/NEMS) because such resonators can perform a dual function of detection and sensing. Recently developed models, based on comprehensive nonlinear vibration analysis and supported by molecular dynamics simulations [147,148], present an important tool in this respect. In this context, we also mention recent work [149] where the focus has been made on mass sensing applications while using boron nitride nanotubes (BNNTs) and carbon nanotubes (CNT) as the strongest lightweight nanomaterials. Importantly, with their structural stability and chemical inertness, BNNTs have nontoxic properties, and their applications in health, environmental, and biomedical areas look quite promising. On the other hand, it should be noted that in our quest to better understand their properties and to improve their performance, the design of BNNT-based sensors leads to nonlinear mathematical models. Note also that in many NEMS and various quantum shuttling systems proposals, there is a demand for better models describing quantum transport and often characterized by rich nonlinear dynamics [150]. Furthermore, given millions of atoms in a typical NEMS, their modelling (and ultimately control) is a computational challenge where atomistic methodologies, including ab initio calculations, MD and Monte Carlo simulations, are normally nonviable. Hence, various multiscale modelling strategies have been applied in such cases. Some authors group them in three categories such as (a) direct coupling (e.g., by the decomposing physical domain and imposing interface coupling), (b) top-down (e.g., quasicontinuum, the bridging scale and heterogeneous methods, etc.), and (c) bottom-up (e.g., coarse-grained MD, multigrid bridging, etc.) approaches [151].

In strain-sensing CNT applications, coupled multiphysics models are also essential. Along with carbon single-walled nanotubes (SWNTs), the use of multi-walled carbon nanotubes (MWCNT)-based sensors for strain-related applications has continued to grow. Since the improved properties of flexible MWCNT-based sensors result from the improved characteristics of these materials (such as electrical, mechanical, thermal), coupled models in this area appear naturally. From the implementation point of view, such sensors can be deployed not only in the pure form but also in various composite forms, e.g., via conjugations of MWCNTs with polymers and other conductive nanomaterials. Such composite forms allow us to obtain devices with better sensitivity, robustness, longevity, as well as to benefit from enhanced structural stability properties [152]. The importance of the electro-mechanical response, as well as piezoresistive properties, of conductive CNT-based composite strain sensors, cannot be overestimated. Here, coupled models also become indispensable, allowing for analysis of such response by accounting for electromechanics, as well as for other characteristics such as CNT dimensions, interphase, nanotube waviness, and dispersion state. The design optimization of highly sensitive CNT-based composite sensors [153] can be assisted by computational modelling that can evaluate their key characteristics (e.g., percolated conductive networks, CNT contents, and orientations, etc.). Additionally, as we already mentioned above, piezoresistivity in electrical-resistance-based strain sensing is fundamental. Since in this case, we deal with an electromechanical effect characterized by the reversible change in the electrical resistivity with strain [154], the role of coupled models in this field is indeed paramount.

Among other factors, this is also revealed by the fact that microstructural evolution has to be often accounted for, in particular when irreversible resistivity changes are added to the reversible change at lower strain, resulting in a stronger piezoresistivity. However, even without irreversible resistivity changes, the piezoresistivity, frequently characterized by the gage factor, needs better quantitative estimations [154] as it is a key effect that enables structural materials to be self-sensing.

In the class of low-dimensional nanostructures, zero-dimensional structures have also been receiving growing attention in the development of strain sensors, with various proposals put forward over the last decade. Examples of quantum strain sensors include those based on a strain-driven transition from a quantum dot with inverted bandstructure and robust topologically protected quantum edge states to a normal state without edge states in the energy gap (e.g., [155]). As we already emphasized in Section 2, the knowledge of bandstructure in these problems is critical which, in its turn, requires the development of coupled models. Indeed, the properties of quantum dots (and other low-dimensional nanostructures) are dependent on both geometry and applied strain. It should also be noted that a large on/off ratio of conductivity across the quantum dot (due to the presence/absence of edge states) can, in principle, be controlled with different methodologies (e.g., by adjusting the number of conduction channels in the source-drain voltage window). While in the sub-section that follows, we will mention other methodologies, here we point out that the modelling approach based on multiband k·p Hamiltonians will naturally lead to a coupled model. The resulting coupled system of partial differential equations appears to be an especially efficient tool for the analysis in such situations, providing an excellent trade-off between accuracy and computational cost.

The accuracy of the k·p approximation is determined by the choice of the set of basis functions that span the functional space where we seek the envelope function. Maitaining a practical balance between the physics of the problem and its computational complexity does not usually lead to the choice of “N” in (5) higher than 8, which is the case, for example, for wurtzite semiconductors. The resulting model with the 8 × 8 Hamiltonian is based on six valence subbands and two conduction subbands, accounting for spin up and down situations and requires the solution of the corresponding PDE eigenvalue problem. The resulting problem is solved numerically, e.g., with finite element method (FEM). For the given approximation, stable FEM implementations, free from spurious solutions, have been developed [156,157]. As a result, the band structure and optical gain of low-dimensional nanostructures can be determined using the 8-band k·p model. This model provides an excellent approximation for most practical cases describing electron, heavy-hole, light-hole, and spin-orbit split-off bands around the Γ point of the Brillouin zone, while treating all other bands as remote bands [5,158]. In this case, the wave function of a state *n* with energy $E_{n}$ can be represented by a linear combination of the eight Bloch parts/states ($u_{i}$) weighted by the respective envelope functions ( $\phi_{i}$):

(7) $$\Psi_{n}=\sum_{i=1}^{8}\phi_{i}u_{i}.$$

Then, electron-hole states are the eigenstates of the 8-band envelope function equation given by:

(8) $$H\Psi =E\Psi ;\;\Psi ={\left(\Psi_{S}^{↑},\Psi_{X}^{↑},\Psi_{Y}^{↑},\Psi_{Z}^{↑},\Psi_{S}^{↓},\Psi_{X}^{↓},\Psi_{Y}^{↓},\Psi_{Z}^{↓}\right)}^{T}.$$

In the above model, $\Psi_{X}^{↑}=\left(|X>|↑\right)$ is the wavefunction component corresponding to the *X* Bloch function of the valence band with the spin function of the missing electron “up”, the subindex “*S*” denotes the wavefunction component of the conduction band, and so on, and *E* is the electron–hole energy. Finally, a generic representation of the Hamiltonian in the k·p theory can be given as:

(9) $$H\equiv H^{\left(\alpha ,\beta \right)}\left(\vec{r}\right)=-\frac{h^{2}}{2m_{0}}\nabla_{i}H_{ij}^{\left(\alpha ,\beta \right)}\left(\vec{r}\right)\nabla_{j}.$$

In the above representation, *H* is defined by the standard Kohn-Luttinger Hamiltonian or by its refined version based on the Burt-Foreman correction taking into account the properties of degenerate valence states in an electric field. From a physical point of view, this representation acts for the kinetic energy plus a nonuniform potential field and other effects contributing to the total potential energy of the system. The superindices (α,β) denote a basis for the wavefunction of the charge carrier [5].

Such coupled models are important in all straintronics and spintronics applications [66,67,159,160,161,162,163,164,165,166,167], and they have frequently been considered as a design element of several important classes of strain sensors and in related lines of research. For example, topological insulators are natural candidates for low-power spintronics because of their intrinsic dissipationless feature. It is possible, for example, to combine the mechanical strain and the giant magnetoresistance of a ferromagnet junction of the topological insulator to construct a novel straintronics device with a robust strain-controllable magnetic switch [168], and even tune magnetic and electronic properties mechanically [169]. These recent studies of strain-sensitive giant magnetoresistance responses, along with other phenomena, have indicated that these types of devices have a great potential for low-power nanoscale strain sensors and other sensing applications. Many other phenomena are getting increasing attention of the modellers, including those connected with with time-dependent transport, inelastic transport, as well as slow light, which was reported earlier in semiconductor heterostructures (e.g., [170] and references therein). Models in this area have proved useful, e.g., in speeding up LiDAR (Light Detection and Ranging) sensors developments and other applications [171].

### 4.2. Properties of Nanostructure-Based Sensors, First-Principles Approaches, and Bandstructure Calculations

In Section 2 we emphasized the importance of the bandstructure knowledge in designing nanostructure-based sensors. Apart from the first-principles calculations, we have a range of computationally feasible numerical methodologies that can be used for bandstructure determination. From a modelling point of view, such methodologies always require a choice of the basis functions, and the case when we deal with Schrodinger-type equations is not an exception. This choice is usually done based on empirical or semi-empirical types of arguments, e.g., on atomic-like or plane-wave representations or Bloch states. The result is one of the commonly used bandstructure calculation methods, the tight-binding, pseudopotential, or the k·p method. Based on the latter method, a robust framework has been developed to incorporate coupled effects, which are pronounced on different scales, via an appropriate construction of multiband Hamiltonians [61]. Unlike ab initio or first-principles methods, this framework provides both, the feasibility of dealing with the multiscale nature of problems in these application areas and computational efficiency. At the same time, the predictive capabilities of such methodologies as DFT, based on the quantum-mechanical description of interacting atoms and electrons, may remain the method of choice when the corresponding calculations are numerically possible for the given system. Moreover, such first-principles electronic-structure methods as DFT can frequently be combined with classical molecular dynamics (MD) to provide an approximation to the system atomic dynamics and important insights into its properties. Depending on a specific situation, the MD approximation with its massive, point-like nuclei and empirical-potential-derived Newtonian forces acting between them may or may not be sufficiently accurate. In the latter case, a modification of the classical MD can be attempted, where we will still treat atomic nuclei as classical particles, but the forces acting on them will be treated quantum mechanically and derived from electronic-structure calculations. While this modified methodology provides an improvement in accuracy compared to the classical MD and the quantum mechanical treatment of only electronic subsystem can often be justified (via the mass difference between electrons and nuclei), nuclear quantum mechanical effects can still be relevant and applicable [172,173] (additional examples are provided in Section 5.1 in the context of rapid biosensing). Since the latter is already the case even for simple situations involving hydrogen atoms, further corrections via ab initio path integral approaches might be attempted. However, such approaches are not always computationally feasible for real systems. There are many arguments in favor of this methodology, in particular when temperatures are comparable with the highest vibrational level in the system considered, but one has to accept the assumption that (non-quantized) vibrational degrees of freedom follow a Boltzmann statistic as a consequence of the Newtonian consideration of nuclei evolutions [172]. In data-driven environments, with the development of novel approaches based on machine learning, computational procedures for such ab initio (MD) path integral approaches and their algorithmic implementations can be substantially advanced [174].

Any design of sensors, which includes their functioning in a real system, is a multiscale problem. As a result, we inevitably have to deal with (multiband) Hamiltonian approximations augmented by the information coming from larger (system’s or device) scales. Hence, the next section is devoted to the modelling aspects of low-dimensional nanostructures and smart materials at such scales which allow their integration with the approaches described above.

### 4.3. Continuum-Mechanics Coupling, Reduced-Order Methods, Coarse-Graining, and Other Numerical Procedures

In the prediction and control of the properties of low-dimensional nanostructures, it is essential to account for coupled effects that are acting at larger than quantum mechanical scales. Such effects should be integrated within the bandstructure calculations of these systems as it has been demonstrated for quantum dots in [61,175] under consideration of coupled magnetic, thermal, and electromechanical fields, as well as the quantum dot underlying surface/interface. The developed framework was extended to other classes and low-dimensional nanostructures and also used for graphene quantum dots [63].

Similar arguments based on the importance of coupled effects are applied to other low-dimensional nanostructures, including those that came to light for sensor applications more recently, e.g., graphene nanoribbons [57,163,164,165,176,177,178,179,180,181]. It is important to emphasize that some of the developed models have to deal with nonlinearities, including strain nonlinearities and non-trivial situations in constructing multiband Hamiltonians [28,30,156,182,183,184,185,186,187], which are essential in the design of sensors based on such low-dimensional nanostructures. At the initial stage of the analysis, first-principles methods can provide good guidance, while subsequent optimization of design characteristics and properties in data-driven environments may require data assimilation technique and machine learning algorithms [68,69,174].

For smart materials such as SMAs, the applications we are interested in require knowledge on the dynamics of the underlying nonlinear physical processes, in particular, coupled thermomechanical interactions and microstructure evolution [188,189]. The main methods for model developments in this area are based on the phase-field ansatz of the Landau theory of phase transitions [190,191], as well as on reduction-order techniques, supplemented by efficient numerical procedures [192,193]. Given the importance of geometry considerations, already emphasized in Section 2, the latter may include the procedures which have no geometric approximation errors, in particular, isogeometric analysis [194,195]. The developed models have been applied to the studies of shape memory nanostructures such as nanowires [113,114]. Computationally efficient reduced-order models have been developed in this field based on center manifold theory [196,197,198] which provides a systematic approach in constructing increasingly accurate approximations with respect to both spatial and temporal parameters.

Finally, we mention efficient coarse-graining models [199,200,201,202,203,204] that have been developed for nucleic-acid-based nanostructures discussed in detail in Section 5.2.

## 5. Further Characteristics and Areas of Applications

We start this section with the recent progress in developing sensors based on graphene allotropes, paying special attention to CNT-based sensors, followed by a discussion of nucleic-acid-based sensors and their applications.

### 5.1. Carbon Allotropes and Sensors

First, we note that among carbon allotropes, carbon-nanotubes have played an especially prominent role in the development of sensors, in particular for biomedical and environmental applications. It has been known for quite some time that CNTs can be used as scaffolds for the immobilization of biomolecules at their surface. They possess exceptional characteristics and properties, including physical, chemical, electrical, and optical. As a consequence, CNT-based biosensors have been an active area of research for a long time [205]. If we take, for example, materials for the transduction of signals associated with the recognition of analytes, metabolites, and disease biomarkers, CNTs are still considered one of the best-suited classes of such materials. This comes, however, with a range of challenges. Among them is the accurate description of CNT properties, their functionalization, biocompatibility, cellular uptake, and toxicity issues, all of which have been approached by various modelling techniques. The analysis of CNT-conjugates, engineered for biosensing applications such as detection of disease biomarkers, also remains a quite challenging task.

New advances in nanomedicine which are heavily relying on LDNs, have appeared as a powerful instrument in overcoming various diseases, as well as the coronavirus pandemic [206]. Since the SARS-CoV-2 pandemic has started, a new demand for the development of rapid testing tools has led to a considerable amount of innovative research on CNT-based sensors for biomedical applications. In particular, the description of near-infrared nanosensor’s stability and sensing mechanisms in the pursuit to preserve sensing response in saliva and viral transport medium was a subject of recent publications (e.g., [207]). Another important direction has been connected with adding functionality to CNTs via chemical modifications and the development of nanosensors based on carbon nanotubes, graphene technology, and plasmonic resonance effects [208]. A review of recent technologies and sensor-based techniques for the diagnosis of COVID-19, including details on graphene-based FET and other innovative sensors for rapid testing, can be found in [209]. These new lines of research could potentially greatly assist in the detection of viral pathogens with opportunities for rapid testing at times of pandemics, allowing to monitor and mitigate the spread of disease outbreaks [208]. Again, from the modelling perspective, the associated models have to couple the physical, optical, and electronic factors as being decisive for the successful development of testing devices and carbon nanomaterial-based sensors. Concurrently, the development of such models for nanosensors is essential due to their great potential in a wider range of applications, including diagnostic medicine in general and enabling early identification of disease without reliance on observable symptoms. A long-time objective in this field would be incorporating both diagnostic and immune response functionalities while transmitting data to allow for monitoring of the sensor input and response. In the meantime, much of today’s research is concerned with the immediate diagnostic capabilities of nanosensors, intracellular implementation protocols for nanosensors synthesized with biodegradable polymers for drug deliveries and treatments, and detection of various contaminants in the body or organ implants. In this sense, the current research that is taking place in the context of biosensors for SARS-CoV-2 detection could also be useful for the above tasks, given the analysis of the properties of nanomaterials targeted for this, including graphene, molybdenum disulfide, carbon nanotubes, and quantum dots due to their unique sensing capabilities [210,211]. More generally, graphene and its derivatives provide a very important source for sensor developments. We consider [212,213] to be excellent initial references on this topic. They cover electrochemical sensors based on 3D graphene materials, graphene-oxide sensors, graphene-based fexible sensor and actuator technologies [212], as well as an impressive range of graphene-based biosensor developments and applications, including biomedical, agro-defense, food safety, pesticides electroanalysis, rapid in-field detection of food toxicants and environmental pollutants, together with several computational frameworks, including the finite element method [213].

The coupled field modelling framework was developed for the evolution and self-assembly of an array of CNTs in earlier works [214,215,216,217,218,219,220,221,222,223,224,225,226]. These developed coupled multiphysics models for CNTs have a number of existing and potential biomedical applications, including imaging. Nowadays, nanosensors represent a central tool in many approaches to molecular recognition, not limited to imaging, as they include also such areas as drug delivery systems and phototherapy. One of the main reasons behind a wide range of applications of CNTs in these areas lies with the fact that single-walled carbon nanotubes (SWCNTs), for example, fluoresce in the near-infrared part of the spectrum, indicating that biological objects can be studied with high efficiency. Further, the biocompatibility of SWCNTs, as well as their unique optical properties, where fluorescent nanoparticles’ emission signal can serve as an optical reporter for a location or environmental properties, lead to the multifaceted applicability of SWCNT sensors in different biosystems, ranging from single-molecules to in-vivo sensing and providing a deeper insight into many biological phenomena and processes [227]. Clearly, the importance of molecular recognition of small molecules (e.g., DNAs, RNAs) proteins and viruses goes well beyond biomedical and healthcare applications, aiming at improved clinical diagnostics and treatments, as it also includes other diverse areas such as environmental and food sciences, to name just a few [147,148]. In a generic sense, the success of the sensors in these fields is based on the combination of recognition capabilities with signal transduction, contributing to their sensitivity and selectivity. These two functionalities of sensors are in the heart of many applications we are discussing here: (a) target recognition may include molecular imprints, nucleic acid sequences, etc., whereas (b) signal transduction can be achieved, providing an optical indication of target binding with one of the available techniques, e.g., labeling with fluorescent dyes or gold nanoparticles, etc. As we already exemplified earlier, a wide range of nanostructures and materials, like nanowires, quantum dots, and various smart materials, demonstrate their potential in high sensitivity and selectivity, and can be advantageous in sensing At the same time, this section highlights also that the use of CNTs as a nanosensor basis in many applications, and in particular in biomedical and environmental areas, is not accidental and comes as a result of their coupled properties (thermo-electromechanical, mechanical durability), an abundance of options for further functionalization, doping, and chemical modifications [227]. Some applications of MWCNTs in sensing have already been discussed in Section 4.1, and over recent years, new applications of MWCNT-based sensors have been actively developed in biomedical fields, in particular in modern medical equipment for health diagnostics and treatment (e.g., [228]). These developments are closely connected with the progress in flexible wearable electronic devices (see also Section 5.3).

While continuum-type models remain a computationally efficient and practical tool in these fields [147], in a number of cases more refined models such as those based on molecular dynamics simulations are required to complement such models [148,229]. Among other applications, this includes the development of nanoelectromechanical systems working in the terahertz range, where the employment of CNTs has opened up new areas related to drug delivery, atomic transportation, and detection of atoms and molecules.

As for other functionalities, CNT-based nanosensors have been demonstrating excellent capabilities in differentiating distinct gas atoms, showing high sensitivity, and being able to distinguish, for example, noble gases at the same environmental temperature and pressure. Different computational strategies have been used for the analysis of such sensors. For example, in [229], a combination of molecular dynamics and a continuum mechanics shell model was used to analyze the characteristics of wave propagation in such CNT-based sensors, providing new insight into the inertia and strengthening effects induced by the gases on CNT wave characteristics. It should also be noted that CNT-based field-effect transistors (FETs) provide novel sensing device configurations that have attracted interest not only in healthcare, biomedicine, and environmental science but also in other areas of applications such as food quality monitoring. These configurations have led to the novel designs of and new proposals for extraordinary chemical and biological sensors [230].

Several interesting ideas useful for sensing applications are coming from twistronics, and in particular from explorations of various non-trivial effects in graphene superlattices and other graphene-based structures [231,232]. Generically, twistronics can be considered as an electronic platform offering a novel approach in device engineering. In the context of sensing, it opens the road to single photon sensing developments [233].

Finally, increasing recent interest in nanoribbons in general [57], and graphene nanoribbons in particular [181], has been driving many innovative sensor applications. ab initio calculations of such nanoribbons, often combined with advanced machine learning techniques, are providing guidance for novel designs [68,69,181]. Along with graphene nanoribbons considered increasingly frequently as a platform for developing advanced biosensors [234], there have been also an increasing interest in graphene-oxide (GO) structures, including superlattices, in particular for biochemical sensors [235].

### 5.2. Nucleic-Acid-Based Sensors and Related Applications

**Figure 6:**
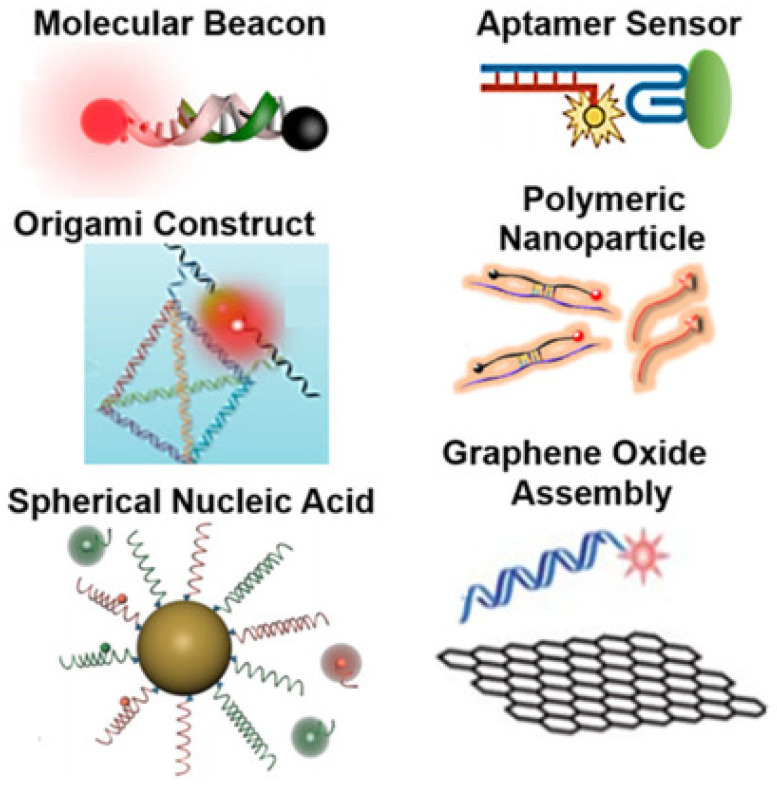
Schematic of some of the activatable optical sensors based on nucleic acids. (This figure is reproduced with permission from [236]).

As we saw in the previous sections, there are innovative designs of sensors based on low-dimensional nanostructures, such as nanowires, for detecting tiny bio-particles such as DNA, RNA, proteins, viruses, and bacteria [147,148]. In the analysis and modelling of such sensors, a combination of continuum mechanics methods and molecular dynamics simulations are applied to quantitatively characterize frequency shifts due to the added bio-object mass. At the same time, there is also a wealth of developments in designing nucleic acid sensors in recent years. As their name suggests, these sensors are composed largely of nucleic acids, and hence are biodegradable, thus, posing minimal to no risk of toxicity within the human body as compared to the chemically synthesized sensors. Moreover, the recognition capabilities of nucleic acid sensors are largely predictable and tuneable due to their highly defined base-pairing assembly that allows precise programmability of their sensing performances. Nucleic acid based sensors are highly versatile and can be easily targeted with nucleic acids and proteins for theranostics purposes. Furthermore, the secondary structures of nucleic acid sequences with certain target compounds (e.g., mRNA or peptides) are highly predictable, owing to their well-characterized hybridization, thus allowing for specific design with greater thermodynamic stability. For e.g., hairpin-shaped molecular beacon probes undergo linearization after nucleic acid recognition, resulting in the changes in fluorescence signal emissions. Recently, nucleic acids sensors have also been prepared through self-assembly of multiple nucleic acid sequences which is termed as DNA origami. Nucleic acid probes can also be loaded on biodegradable nanoparticles that can be further utilized for wide range of extracellular, intracellular and in vivo applications [236]. Figure 6 presents some of the activatable optical sensors based on nucleic acids. Such DNA/RNA biosensors will be the main subject of this section where we also touch upon recent developments in atomistic-to-continuum models [237]. The information extracted from DNA/RNA sequences has led to much innovative research in disease diagnostics and treatments, biomedicine, and environmental sciences. This, in its turn, brought about the interest in devices, engineered from molecular probes and nanomaterials, as the basis for DNA/RNA biosensors. The goal in this field has been largely driven by the desire to achieve improved characteristics in sensitivity, throughput, cost-effectiveness, portability, and flexibility. This research has included also microfluidics applications in creating the lab-on-a-chip paradigm for integrating sample preparation with biosensors to realize point-of-care (POC) diagnostics. New sensor-based technologies have been developed for DNA sequencing techniques where DNA sensors based on graphene are playing an important role. For example, one of the directions that have been pursued in this field is pertinent to graphene-based nanopore devices with nanopores formed at the edge or in the center of graphene nanoribbons [238].

Among other applications, we would like to mention the rapid identification of infectious agents. This area is critical for the management of infectious diseases by providing the possibility of prompt therapeutic intervention. In this context, nucleic acid-based detection can bring forth higher sensitivity and shorter turnaround time (see, e.g., [239] and references therein). Furthermore, enhanced sensitivity and multiplexing capabilities can be achieved by the coupling of electrochemical biosensors with nanoparticles. Moreover, a substantial amount of the reported research has been carried out on the use of semiconductor quantum dots conjugated with nucleic acids for inhibitor screening of various virus proteins, and on exploring the potential in developing new methods for efficient screening for antiviral drug discoveries.

**Figure 7:**
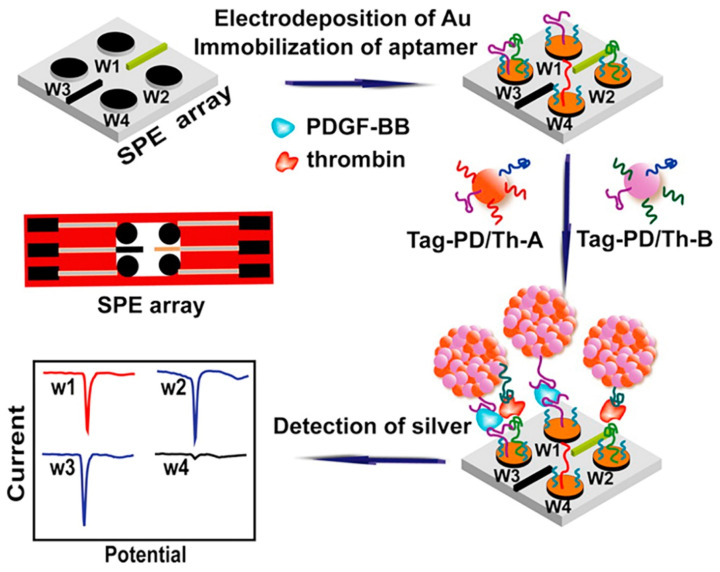
Schematic illustration of the electrochemical aptasensor for multiplex detection of PDGF-BB and thrombin. (This figure is reproduced with permission from [240]).

Nanocluster-based DNA/RNA probe designs are diverse and their scope is indeed impressive. Out of a myriad of applications of such biosensors they can be applied to the detection and monitoring of environmental pollutants, new chemicals, and drugs, including those producing genotoxicity with damages to the genetic information within a cell via mutations ([239] and references therein). As an example, Figure 7 presents the electrochemical aptasensor for multiplex detection of growth factors that regulate cell growth and division, such as the platelet-derived growth factor (PDGF-BB), as well as multifunctional serine proteases that play a key role in haemostasis, such as thrombin. In particular, silver nanoparticles (AgNPs) aggregates were used as tracing tags for PDGF-BB and thrombin through in situ hybridization of DNA was immobilized on AgNPs. Proteins detection was accomplished for PDGF-BB and thrombin by using the gold nanoparticles modified screen-printed electrode (SPE) array chip as sensing platform. The developed biosensor was also successfully tested in the logic gate operation [240].

There is also an increasing interest in self-assembling nucleic-acid nanosensors, with DNA/RNA architectonics assisting in rapid iterative optimization of sensor designs via biocompatible building blocks which self-assemble upon different mechanisms, e.g., analyte detection. This self-assembly is guided by the principles encoded naturally in nucleic acids. An important direction of the research in biosensing (including even sensing of drug release) is related to bioconjugate nano-assemblies which are frequently based on low-dimensional nanostructures such as quantum dots [241,242,243]. Some of the techniques used in such cases, in particular when conjugating proteins to QDs, include bioluminescence resonance energy transfer (BRET). Quantum dot DNA nanosensors have been under very intense scrutiny for quite some time due to the importance of rapid detection of DNA in genetic disease diagnostics and many other applications (e.g., [244,245]). Note also that advances in biomimetic materials and semiflexible biopolymers assist in developing novel sensors. In developing this research direction, the models allowing to analyze the properties of such materials, as well as macromolecules themselves (such as DNAs), are very important [246]. It is interesting to record that in these developments, carbon-based nanostructures may serve as interconnects in electrical and biological systems, emphasizing the importance of models for studying properties of carbon-based materials as well [247,248] which we discussed in the previous Section 5.1. In this context, new models that can assist in integrating nanostructures with biological structures are required. This includes, but is not limited to, the integration of CNTs and biomolecules such as peptides, proteins, antibodies, DNAs, RNAs, etc. along with the design of new sensors based on these concepts [249,250,251,252].

Moreover, there are also a number of proposals for hybrid nanostructures where various design and screening strategies can lead to a myriad of RNA-DNA hybrid nanostructures that can be used as programmable platforms for applications in sensing, molecular recognition, protein interaction, and catalyst research [253]. Given the capabilities of nonlinear responses of such nucleic acid biosensors over a wide dynamic range, refined modelling techniques such as those based on MD simulations are required along with the development of efficient coarse-grained procedures [199,200,201,202,203,204,254]. In this way, properties of associated nucleic acid nanostructures such as RNA nanotubes have been studied in detail, including their behavior in fluids.

Equally important designs are based on DNAs. Indeed, given the significance of protein-DNA and drug-DNA binding, the development of novel nanosensors in the same length scale as DNAs has been an active area of research. The current state-of-the-art in this field includes several nanosensor developments. Some of them include nanosensors for detecting metal ions and pathogens, including various bacteria and viruses. This detection is based on utilizing techniques for high-throughput screening technologies such as surface-enhanced Raman spectroscopy (SERS), colorimetric, dynamic light scattering (DLS), as well as fluorescence and electrochemical detections, to name just a few [255]. These new developments greatly assist not only drug discovery methods, but also mass screening which is critical at times of pandemics such as SARS-CoV-2.

**Figure 8:**
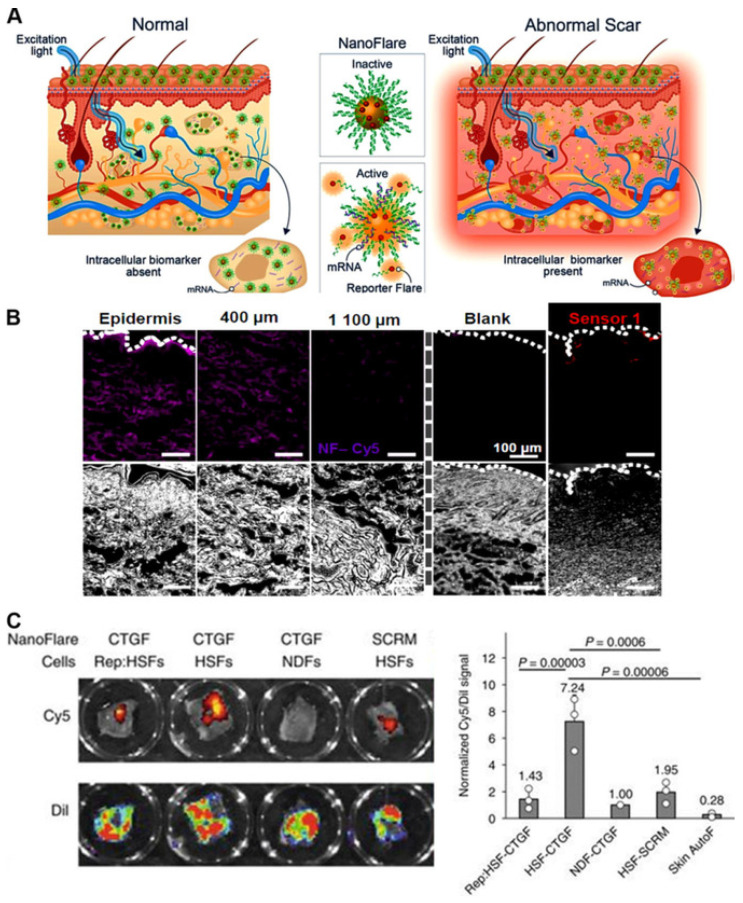
(**A**) Non-invasive detection of an abnormal scar through topical application of Au-based NanoFlares. (**B**) Cream-formulated NanoFlares could penetrate the skin barrier to reach a depth of 1.3 mm in human skin. (**C**) Signal fluorescence restoration then revealed the presence of HSF cells, with significantly stronger signal than normal dermal fibroblasts (NDFs) or RepSox-treated HSFs. Scale bar: 100 μm. (This figure is reproduced with permission from [236,256]).

Figure 8 presents the in vivo applications of nucleic-acid-based nanosensors in non-invasive diagnosis of scar type and severity. In particular, the expression of connective tissue growth factor (CTGF) was measured as a visual indicator of hypertrophic scars and keloids utilizing the imaging nanoprobes for the live-cell detection of intracellular messenger RNA (mRNA) (also known as NanoFlares, i.e., with fluorescence flare strands that were detachable upon mRNA hybridization (Figure 8A)). NanoFlare were applied non-invasively through topical application of Aquaphor (a common skin moisturizer) on the skin of live mice, rabbits, and ex vivo human skin models (Figure 8B). Transepidermal penetration of NanoFlare enabled the distinction of normal fibroblasts from hypertrophic and keloidal fibroblasts, along with the detection of changes in CTGF expression (Figure 8C) [236,256]. We also note that dynamic properties of nano-bio interfaces have long been studied for their direct applications [257] and further potential in such areas as DNA biosensors and microarrays due to the possibility of hybridization even at very high DNA surface coverage. What is more, it is realizable to construct such interfaces reflecting or mimicking the characteristics of their biological counterparts. Apart from sensing and biomolecular detection, these new systems are expected to provide improved capabilities in such areas as gene therapy, neural prosthetics, and regenerative medicine, to name just a few.

Bionanotechnology plays a critical role in designing novel electrochemical sensors. In particular, hybrid systems based on a combination of biomolecules and inorganic nanoparticles have been used as a platform for interfacing biological recognition and electronic signal transduction, allowing innovating designs of high sensitivity bioelectronic devices [258,259]. Sensing technologies are also assisting important techniques based on biological processes such as RNA interference, gene silencing, and others [260]. We will mention here, as an example, compressed sensing methodologies which are used in RNA interference (RNAi), various specific problems in metagenomics, and in genomics in general [261]. Moreover, low-dimensional nanostructures such as quantum dots are also playing an important role in further developments of these techniques, including gene silencing [262]. Furthermore, in the development of new technologies and techniques for biomedical applications, including those sensor-based, the tools and methods of mathematical modelling are especially important. Some of such techniques aim at fast macromolecule (DNAs, RNAs) sequencing, where electronic transport properties of such macromolecules, which are often in the heart of these techniques, are usually studied via mathematical and computational models [263,264]. It should also be noted that while studying electronic transport properties of macromolecules in the context of sequencing mentioned above, coupling effects become crucial [265] which is also true for novel graphene-based devices for macromolecule sequencing [266]. For the last two decades, the sensing and detection of the state of biological systems and living organisms have remained one of the central research directions in bionanotechnology. This can be done by a variety of methods such as electrical, magnetical, and optical, allowing, in its turn, to revolutionize many areas in materials chemistry and physics [267].

Nucleic acid nanotechnology assists in innovative breakthroughs in sensing, designing DNA/RNA machines, and drug delivery systems [268]. For example, in this latter publication, the authors developed multiplexed sensing platforms for targeted DNAs based on hybrid systems of nucleic acid-semiconductor quantum dots. The underlying mechanism for the amplified detection of DNA targets (carried out via the biocatalytic regeneration of analytes) was relying on the chemiluminescence resonance energy transfer. Other technological developments in this field include a variety of nucleic-acid-based sensors nanopores, and ion channels, while chemical functionalization of DNA allows us to convert static nucleic-acid structures into dynamic nanomechanical sensors [269].

Concluding this sub-section, we would also mention a wide range of biosensors based on other ideas such as ion-channel switches (ICS) and artificial membranes. Several tethered-membrane-based sensor platforms exist for the development of such biosensors (see details, e.g., in [270] and references therein). For these types of biosensors and associated artificial cell membrane systems [271], there are many multiphysics dynamic models developed in the literature, ranging from ab initio MD, coarse-grained models, as well as continuum approximations. This also includes the so-called reaction-rate-constrained models for the ICS biosensors where probabilistic approaches based on hidden Markov models have also been developed [270]. Starting with FRET-based biosensor systems [272], the development of various types of biosensors with low-dimensional nanostructures such as quantum dots continues driving much of the innovative research in this field [273].

### 5.3. Environmentally-Friendly and Other Innovative Technologies

Sensor developments are among the important directions in environmentally-friendly and innovative technologies. Let us have a look at the green energy sector first. Here, the importance of quantum heterostructures for high-efficiency photovoltaic cells, as well as for photovoltaic systems in general, cannot be overstated. In developing many photodetectors and photovoltaic systems, nanowires and nanotubes are traditionally playing a prominent role [274]. In the meantime, the modelling and analysis of photovoltaic stability greatly assist in designing better solar cells, field emitters (mentioned also in Section 5.1 in the context of CNTs), and other devices. While photovoltaic sensors can be viewed as a special type of photodetectors, the rapid development of renewable energies with photovoltaic systems’ interconnections to the grid at competitive cost [275], make them one of the frontrunners in the green economy, energy harvesting systems, as well as for other applications such as the “internet of things” [276]. Many innovative proposals have been put forward along these lines which also included devices developed on the basis of the van der Waals heterostructure technology, e.g., graphene/boron nitride/graphene heterostructures. It was observed, for example, that for graphene/boron nitride/graphene heterostructures, the electronic properties can be efficiently controlled by a combination of the selection of materials in the stack and the additional tuning through adjusting the built-in strain and relative orientation of the component layers. This technology assisted in our better understanding of the metal-insulator transitions and Coulomb drags and led to the realization of a series of functional devices, including photovoltaic sensors [277]. Heterostructures, which we discussed in Section 2, are also critical for a range of energy storage and conversion solutions, solar cells, supercapacitors, lithium batteries, thermoelectric devices, catalysis, and fuel cells to name just a few. In this context, important research has been carried out on the design, synthesis, and applications of one-dimensional nanostructured surface heterostructures which have also applications for sensors [278]. A special type of gas sensors, as well as hydrogen sensors, are important for the green economy too. For hydrogen sensors, their design is connected with a number of challenges, one of which is to have a nanostructure in place that would allow amplifying changes in light transmission after hydrogen absorption. New progress in this direction has been recently reported in [279]. In Section 2, a number of examples were given for the application of zinc oxide in sensing applications. Such applications have traditionally covered a wide spectrum of areas such as energy, gas, chemical, and biological sensors [280]. For advanced energy applications, for example, probabilistic computational approaches, including those rooted in Bayesian statistics and parametrization, have been growing in popularity. Moreover, in Section 2 we also already emphasized the importance of gas sensing technologies in the context of environmental applications. Now, we would like also to add to this the importance of the development of advanced models for nanogenerators for self-powered gas and chemical sensing, including piezoelectric and triboelectric nanogenerators, a wide range of nanogenerators based on nanowires, and others. The sensors designed with such nanogenerators not only substantially reduce power consumption compared to conventional sensors, but also they have serious advantages in their applications for wearable devices because they require less space for integration [281]. In pursuing this innovative technological direction, ZnO-based heterostructures [282,283] and models for studying their properties [34,284] are playing a foremost role, along with their applications for renewable and sustainable energy sources where these and other nanotechnological advancements are critical [285]. Of course, when we are talking about the latter areas of applications, we cannot leave out graphene-based materials [286] with their intriguing coupled electrical, optical, mechanical, thermoelectric, and magnetic properties. Moreover, it is unquestionable that sensor technologies in sustainable development are indeed pivotal [287,288].

**Figure 9:**
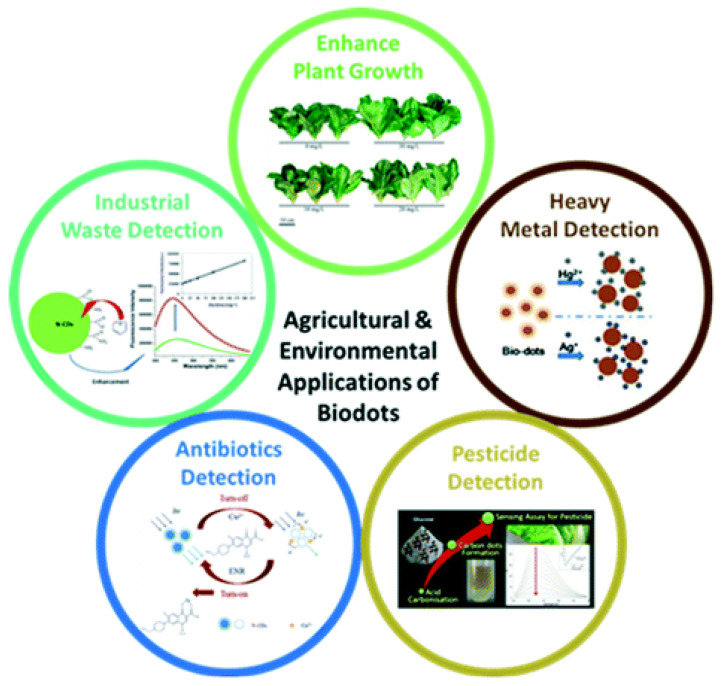
Agricultural and environmental applications of biodots for enhanced plant growth, heavy metal detection, pesticide detection, antibiotics detection, and industrial waste detection. (This figure is reproduced with permission from [292,293,294,295,296,297]).

Many environmental issues are addressed with the assistance of sensor technologies where nanomaterials are playing a crucial role. This includes low-dimensional nanostructures, studies of quantum dots for identifying biological materials, nanoparticles combined with polymers, composites, and other receptor molecules that change color when contacted by analytes (e.g., by toxic gases). An example of environmental (and agricultural) applications is given in Figure 9. Specifically, we see the application of bioinspired carbon dots (biodots) in these areas. Such biodots can be used in ultrasensitive fluorescent sensors for detecting water pollutants or industrial wastes such as heavy metals, pyridine compounds, pesticides, etc. Some effective fertilizers for plant growth enhancement can be designed based on such carbon dots. Continuing with the environmental domain, we also note many innovative designs of humidity sensors that have been proposed on the basis of CNTs, utilizing their excellent properties (electrical, mechanical, chemical). Such sensors have been analyzed recently in [289], based on the underlying mechanisms and types of CNTs (e.g., semiconducting s-CNTs, metallic m-CNTs, depending on the chiral vector during CNT growth). Markedly, the implications of this work go well beyond environmental sciences and include industrial, agricultural, and medical applications, along with the creation of smart wearable electronic devices. Unquestionably, nanomaterials-based electrochemical sensors are not limited to CNTs only, and the area of applications of such sensors is growing. For example, such sensors can be used for serotonin detection which is important for brain and nervous system cells to communicate with each other, given that serotonin is one of the key hormones that is responsible for our mood, helping also sleeping, eating, and digestion [290]. Possibilities for the design of such sensors include modifications with CNTs, graphene oxide, silver-silver selenite nanoparticles, zinc oxide, to name just a few. Nanocomposite sensors based on conductive polymers, which we shall mention again shortly, are also important in this area. Additionally, the pharmaceutical industry and new biomedical and environmental challenges faced by humans lead to a surge in the development of nanomaterial-based electrochemical sensors and biosensors for the detection of pharmaceutical compounds. Notably, recent reference [291] has focused on such developments for the following compounds: anti-inflammatory, anti-depressant, anti-bacterial, anti-viral, anti-fungal, and anti-cancer drugs.

Evidently, these developments are not limited to pharmaceutical production, drug delivery, and specific technologies (e.g., to track patient overdosing, to monitor ambient water sources or wastewater for pharmaceutical pollutants). The advanced sensing of pharmaceutical compounds is needed on a global scale to safeguard human health and ecosystems. Other types of electrochemical sensors have also been playing an important role in addressing such environmental issues as nitrate detection which can be done effectively over a wide range of environmental samples. The choice of nanomaterials in the design of such sensors is very important. Recent studies have demonstrated the effectiveness of metal/bimetal/metal oxide nanoparticles and graphene derivatives, in addition to carbon nanotubes and fibers [298]. It should also be noted that for modifying electrodes in enzymatic and non-enzymatic electrochemical nitrate sensors, conducting polymers represent another important class of materials. The idea of conductive polymers has been extended to conductive networks which, together with metamaterials, today represent an important direction of research in sensor technologies. For example, in [299] CNT-based flexible conductive networks for resistive-type sensing applications have been discussed, including recent progress on the formation of different conductive sensing networks within elastic polymers exploring their nanoscale structure. The microstructures of CNT-based conductive networks are quite diverse, including uniform mixing and aligned structures, multilayered and porous structures, nanomesh, wrinkled, and weaving structures. This diversity brings about serious modelling and computational challenges. At the same time, the range of applications of such conductive networks for sensing technologies is ample, including personal healthcare, body motion detection, smart robots, human-machine interactions to name just a few. There are also CNT-based flexible metamaterials for THz sensing that are under active development. Again, the application range of such sensors is huge and also includes environmental issues such as pesticide sensing [300]. Apart from biochemical sensing, surface defect detectors, strain sensing, and wearable terahertz imagers are also often brought up. The rapid development of the field of flexible terahertz metamaterials is frequently attributed to the inherent limitations of metal-based metamaterials and many advantages of CNTs come in handy here too. New flexible terahertz metamaterial sensors can be based on subwavelength periodic array structures of carbon nanotube thin films (see also Section 5.1). One of the novelties here lies with the fact that flexible metamaterials can achieve the surface plasmon resonance resulting in enhanced resonance transmission peaks [300]. Plasmonics play a vital role in today’s sensing technologies and their developments. In this context, rapid testing devices have already been mentioned in Section 5.2. In general, localized surface plasmon resonance and surface plasmon resonance remain some of the most widely used refractive index-based biosensing methodologies [301]. The performance of plasmonic sensors depends heavily on the underlying geometry as well as on the nanomaterials employed, bringing serious challenges in their modelling and design similar to what we discussed in Section 4. At the same time, plasmonics provides an important potential route to create ultra-sensitive and long-range sensing networks that can be deployed in a variety of sensing applications, beyond biochemistry. Plasmonic biosensing and low-dimensional nanostructures have formed a strong alliance for quite some time now. At the heart of this alliance is the use of the plasmonic resonance of nanoparticles that allows the creation of ultra-small plasmon sensors for detecting analytes such as biomolecules. This plasmonic resonance is sensitive to the spectral shift which is dependent on the relative size of the biomolecules and the plasmon field (e.g., [302]). Further, plasmonic nanoparticles and superlattices are both important in sensing, as well as their combinations. Indeed, recent efforts have been directed towards assembling plasmonic nanoparticles into ordered 2D- and 3D-superlattices, having in mind applications for plasmonic sensing [303]. Given collective plasmon-polariton modes and other complex dependencies within such plasmonic superlattices, the modelling will continue playing a key role in this field, complementary to experimental investigations. Our review would not be complete if we would not mention new developments in photonic crystal sensors with their types ranging from biosensors and integrated labs-on-a-chip to chemical and mechanical sensors, along with many other applications [304,305]. Photonic sensing platforms have also undertaken intensive developments, where low-dimensional nanostructures such as nanowires have been playing a prominent role [306]. As in other areas we have reviewed here, the role of mathematical modelling, complementing experiments, has been paramount here for several decades now [307], with new ideas at the hybrid photonic-plasmonic interface have been actively explored [308].

Next, we note that while piezoelectrics remain a key material in sensing technologies and their detailed analysis and modelling in dynamic settings, important for sensor applications, have been a subject of many publications (e.g., [309,310,311,312,313]), nowadays there is an important trend towards the creation and usage of lead-free piezoelectric composites with polymeric matrices. Such composites can offer a scalable and eco-friendly solution to sensing and energy harvesting applications and the most recent developments have been reported in a series of papers [143,314,315,316,317,318,319] where the issues of nanoadditions, such as CNTs and/or graphene, their importance and comparisons [320,321], have also been discussed.

In the case of medical diagnostics and treatments, sensors may need to deal with non-Newtonian flows, as it can be the case for Venturi types of channels/devices or other applications pertinent to flow sensing, including problems of fluid-solid interactions [322,323,324,325,326].

As it has been emphasized, much of nanotechnological innovation in the field of this review is coming from the development of nanosensors which allow us to detect and monitor physical and chemical characteristics at the nanoscale. This is often done by measuring such characteristics as movement, speed, volume, concentration, pressure, temperature, electric and/or magnetic forces, etc. [327]. In Section 4.1 we already emphasized the importance of sensors capable of working in extreme environments, pointing out pressure sensors as an example. Clearly, in addition to, e.g., high-pressure sensor applications [328], there are other applications that are worthwhile mentioning here too such as those for chemical, gas, and biological sensing [329]. Moreover, some of the corresponding devices in such cases are nitride-based, providing a very good platform for the applications of surface acoustic waves as well (see also Section 2). Much of the research has been carried out recently analyzing sensitivity and optimizing nitride-based sensors (see, e.g., [330] and references therein). Among other things, this leads to the possibility of identifying specific cells at the molecular level for drug delivery, monitoring the development of particular places in the body, and applying novel nanosensor technology in biomedical, chemical, manufacturing, transportation, and environmental systems. Today, nanosensors play an important role in improving food and environment sectors including food processing, agriculture, air, and water quality monitoring, packaging, and transport. For example, CNT and graphene-based sensors have been designed to detect toxin compounds in food products and milk, where their presence above a threshold level can potentially lead to immunosuppressive effects and other health-related anomalies in humans [331]. While we already mentioned toxicity issues in this section earlier, they continue to be debated in the literature. Indeed, toxicity at the nanoscale and the development of environmentally-friendly technologies often have to be considered hand in hand, even in the context of evolution in the presence of nanoparticles [332]. As a result, the field of sensor developments that use the achievement of nanoscience and nanotechnology is not an exception in this sense [333]. This concerns also the issues of cytotoxicity and bacterial strains [334,335], as well as genotoxicity in the context of low-dimensional materials and systems in general and LDNs in particular [336,337]. Many applications of nanosensors in biomedicine are closely connected with the achievements of wearable electronics in designing wearable sensors and other flexible lightweight devices. Some of this research is connected with the development of strain sensors discussed in Section 4.1. Among these, there are highly stretchable and sensitive strain sensors based on silver nanowires/MWCNTs on hair bands for monitoring various human actions, e.g., swallowing, motions, etc. [338]. In the context of wearable sensors and biomedical electronics, advanced 2D materials such as graphene [339], as well as smart materials [318], become important candidates. While sensors provide a key element to many new technologies, such technologies along with systems biology approaches empower predictive, preventive, and personalized medicine. Among other tools, wearable sensors and dynamic predictive modelling are rapidly moving to the forefront of these innovative developments [340,341].

In recent years, the usage of and comfort with wearable sensors have grown considerably. Moreover, they are becoming increasingly common in clinical trials, with some estimates indicating that around 70% of all protocols will include them by 2025. Given this, regulations and standards in sensor technologies become paramount [342]. Of course, this is particularly true for the healthcare industry, biotechnology, and medicine, while in general, it can be said that such regulations are dependent on the associated risk that, e.g., is being higher for implantable compared to noninvasive devices. Legal issues are coming hand in hand with such risk that also determines the route (via regulations and trials) of the sensor-based device to the market.

Further, we would like to highlight challenges in applications of these sensor-based innovative technologies, such as the size of the device, its ability to flex or bend, its structural properties, capabilities of harnessing data continually, as well as cost and energy consumption [343]. Once again, when we deal with implantable sensor-based medical devices, even evaluating their market potential for end-user implementations is a complex process [344], yet their usage in predictive and personalized healthcare is critical. The latter often comes with other challenges such as harnessing data by patient-centered sensors which in some cases can be done passively in a person’s habitual environment.

There are two persistent trends in future developments and applications of biosensors in the areas mentioned above. On the one hand, they have transformed and will continue transforming clinical trials [345,346,347]. On the other hand, in their implementations and usage, in particular, those advancing smart wearable or implanted technologies, the importance of regulatory, safety, legal, and market aspects associated with these technologies will continue to grow [348].

One additional burgeoning expansion of sensors with rapidly growing popularity that should be mentioned is the Internet of Things (IoT). Any IoT device relies heaving on sensors or sensor networks which play a critical role in this new paradigm where it is possible to exchange data over a network of interconnected elements or nodes [349,350].

Finally, we would like to make a few remarks regarding several other innovative recent studies. Hybrid structures, including van der Waals heterostructures already mentioned above, with different photosensitive nanomaterials and nanostructures, have been explored because of their improved photodetection and modulation efficiency. Many such structures can operate from ultraviolet to terahertz regimes, and investigating them further, the integration of graphene with silicon complementary metal-oxide-semiconductor (CMOS) circuits, the human body, and soft tissues has already been demonstrated [339], opening new opportunities in these areas. Three-dimensional graphene-based wearable piezoresistive sensors have also been demonstrated [351] as promising flexible sensors in human motion detection, health monitoring, electronic skin, and other areas which represent a critical part of future AI system technologies. This includes also transparent, lightweight, multispectral photodetectors mimicking the human visual system which can be constructed based on semiconductor nanowires [352].

## 6. Conclusions

We have considered some of the key aspects of modelling and applications of sensors in chemical, biomedical, and environmental fields, focusing on those based on low-dimensional nanostructures and smart materials. While nanosensors provide many advantages compared to their counterparts based on traditional bulk materials, their design and optimization of their properties are associated with many challenges at the modelling level. In the first instance, one needs to account for the coupling between different physical fields such as thermal, electromechanical, and magnetic, as well as for additional nonlinear and nonlocal effects which can also be important in many applications. Hence, such challenges include the necessity to develop coupled multiscale models and efficient numerical methodologies for their solutions. The efforts directed to combining traditional methodologies, in particular DFT, MD, MC methods, and those based on continuum descriptions, within a unified multiscale framework have been ongoing for several decades. As a result of these efforts, many efficient methodologies have been developed, while the ultimate goal of systematically coupling the atomistic to the continuum descriptions is still connected with a range of fundamental theoretical and practical challenges. In the meantime, given the limitations of first-principles methods at larger scales, the article has emphasized the importance of the development of reduced-order and approximate models. As examples, in addition to more traditional sensing applications, two major innovative classes of sensors have been considered, those based on carbon allotropes and nucleic acids, driven by the achievements in nanotechnology and the field of architectonics of programmable molecules. As for any new technologies and novel applications, much of the innovative sensor developments and associated models, reviewed here, require future work in advancing further the sensitivity, validity, and reliability of sensors and their networks. Consideration of heterostructures has represented an important illustrative example. Moreover, dynamic nonlinear models have also been considered, focusing on the applications of smart materials in sensor technologies. Given the fact that phase transformations play a decisive role in the properties of such materials and their operation, such models account for dynamic interactions between different physical fields and microstructure evolution. Finally, we have also included sensing applications for environmentally-friendly technologies, including green energy and lead-free structures, closing with several examples of wearable sensors important for future AI systems technologies, as well as for other current and potential applications.

## Data Availability

All data generated or analyzed during this study are included in this article.
